# A Novel Therapy for People Who Attempt Suicide and Why We Need New Models of Suicide 

**DOI:** 10.3390/ijerph14030243

**Published:** 2017-03-01

**Authors:** Konrad Michel, Ladislav Valach, Anja Gysin-Maillart

**Affiliations:** 1 University Hospital of Psychiatry, Outpatient Department, 3008 Bern, Switzerland; anja.maillart@spk.unibe.ch; 2 Private Practice, 3400 Burgdorf, Switzerland; ladislav.valach@hispeed.ch

**Keywords:** attempted suicide, suicide, suicide prevention, action theory, narrative interview, psychotherapy

## Abstract

This paper presents a model of suicidal behaviour based on suicide as a goal-directed action, and its implications. An action theoretical model has guided the authors in the development of a brief therapy for individuals who attempt suicide (ASSIP—Attempted Suicide Short Intervention Program). Key elements are an early therapeutic alliance, narrative interviewing, psychoeducation, a joint case conceptualization, safety planning, and regular letters over 24 months. In a randomized controlled trial, ASSIP was highly effective in reducing the risk of suicide reattempts. The therapeutic elements in this treatment are described and possible implications for future directions in clinical suicide prevention discussed.

## 1. Introduction

“*It should be axiomatic that suicide cannot be prevented until it is properly conceptualized*”.

[1]

Suicide is a multifactorial phenomenon, and there are numerous models of suicide and suicidal behaviour, ranging from Durkheim’s anomic suicide to suicide as an outcome of serotonin dysfunction [2,3]. Ultimately, models of suicidal behaviour must translate into projects that are effective in reducing suicidal behaviour. The clinically most prevalent model is the biomedical model, based on the close association of suicide with psychiatric pathology [4]. However, although psychiatric diagnoses are major risk factors for suicide [5], prevention projects aimed at improving the detection and treatment of psychiatric disorders have limited potential to reduce suicidal behaviour on a population level [6,7]. The evidence for pharmacotherapy, and antidepressant treatment in particular, is mixed [8,9,10], with the exception of long-term lithium treatment [11].

We need to look beyond the medical model in order to improve the effect of clinical suicide prevention [12,13,14,15]. A number of psychotherapeutic treatments, including Cognitive Behaviour Therapy (CBT), Dialectical Behavior Therapy (DBT), and Collaborative Assessment and Management of Suicidality (CAMS), have shown a reduction in repeated suicidal behaviour [16,17,18,19], but due to small numbers and the lack of replication studies, the evidence so far is limited [20,21].

## 2. A Tower of Babel Syndrome

“*I got very angry when they kept asking me if I would do it again. They were not interested in my feelings. Life is not such a matter-of-fact thing and, if I was honest, I couldn’t say if I would do it again or not. What was clear to me was that I could not have enough trust in any of these doctors to really talk openly about myself*”.

[22]

A major obstacle in clinical suicide prevention is the fact that many individuals at risk of suicide, males in particular, do not seek help [23]. We asked patients one year after a suicide attempt, who, in retrospect, could have helped to stop them from harming themselves. Twenty percent mentioned relatives or friends, while 52% said nobody, and only 10% mentioned a health professional [24]. Suicidal patients, above all men and the young [25], do not feel that consulting a health professional might be helpful. Most people experience suicide ideation as ego-syntonic, i.e., as something that does not need treatment. They do not feel ill. Even when people do contact health professionals, suicidality is very often not addressed. In a psychological autopsy study of 571 suicides, in which a health care professional had been contacted prior to the suicide, the issue of suicide had been raised in only 22% of the last visits [26]. Eighteen percent of those who had contacted a physician had done so on the day of their suicide, yet even then, the issue of suicide was addressed in only one fifth of these cases. Similar findings have been reported in Australia [27]. As a rule, suicidal persons, even when under medical care, rarely talk about their intentions, nor do they spontaneously mention past suicidal crises [28,29]. Yet, the other side of the problem is that health professionals rarely ask about past and present suicidality [30,31]. In a survey conducted in Bern, 48% of general practitioners were surprised by the suicide of their patients, and 67% said they had no knowledge of their patients’ previous suicide attempts [32]. Consistent with the general communication problem, the adherence to follow-up appointments after a suicide attempt is low, often being no higher than 50% [33], and these patients tend to drop out of treatment prematurely [34,35]. In a UK study, patients who had been hospitalised after a suicide attempt said that they had found nurses and social workers more helpful than doctors [36]. The staff attribute that showed the strongest correlation with help received, was the ability to listen and to express sympathy.

To be open to listen and to be sympathetic with a patient disclosing suicidal intentions, is undoubtedly a difficult task for the clinician. Medical training equips health professionals with the ability to look for pathology and to diagnose somatic and psychiatric disorders, but it does not provide helpful models to understand peoples’ actions. Thus, the patients’ concepts of suicide and the concepts of professional helpers, do not match, i.e., patients and doctors speak different languages. A meaningful discourse needs a common ground of understanding where the two protagonists can meet.

## 3. Understanding Suicide as an Action

“*I messed up the relationships with most of my relatives, and I am in the train of doing the same thing with my wife. Everything wrong, always, always, always. My best wishes to everybody, including me. I do not know where the journey will go, but at least it does not stay in this vale of tears”..... “I always wanted to keep suicide as the last way out. Maybe I didn’t quite mean it when I first tried to kill myself. Next time will only take place when I really mean it*”.

[37]

Suicide is many things—the most obvious facet of suicide is often overlooked: suicide is an action. Actions, according to action theory, are being carried out by agents; that is, by persons who are setting goals, making plans, who are monitoring and regulating their own behaviour, thoughts, and emotions in the pursuit of their goals [38]. Action theory represents the way in which people explain and understand actions, and it uses concepts from the common vocabulary of everyday life, such as needs, intentions, plans, strategies, decisions, choices, success, and failure. Actions are related to a hierarchy of goal-directed systems, which are shaped by a person’s biography. The higher-order developmental systems include long-term goals relating to a person’s life-career or identity, and mid-term projects, typically related to work or relationships [39]. In a case study, we conceptualized suicidal behaviour using an action theoretical model [37]. We argued that suicide emerges as an alternative to life-oriented goals, when, due to adverse life events, a person is faced with a serious threat to important personal higher order goals, such as to be loved, respected, or to be successful. The emotional experience of such a personal crisis is characterized by psychological pain, hopelessness and feelings of shame, a sense of personal failure, of being useless, of being a burden to others, and self-hate [40,41]. Under high emotional stress, long-term life-oriented goals lose their meaning, and the individual becomes subjected to extreme short-term goals. Suicide then emerges as a possible solution to end an unbearable mental state. The disconnect between the wish to die, the action of killing oneself, and the experience of being killed, has also been conceptualized as a distorted goal-directed action [42].

The way in which we make sense of the actions of others and the way in which we explain our own actions, is through stories or narratives. A narrative is a story told to an attentive listener, giving meaning to events in order to explain the inner logic of a specific behaviour or action. A coherent autobiographical narrative in itself creates a sense of self and mastery. It is also the prototype of a shared experience, or joint project, allowing the patient to generate multiple perspectives. “When we are able to formulate the right story, and it is heard in the right way by the right listener, we are able to deal more effectively with the experience” [43]. Patients’ narratives typically contain clear descriptions of goal-directed processes (see quote at the beginning of this section). Furthermore, patients may describe how suicide first—and often repeatedly—became an option in their lives. They may describe how they came to take the first steps towards suicide, deciding on the means of suicide, their fantasies about the results of their action, and maybe also how they were interrupted in their preparations of the suicidal act. Most patients describe intensive monitoring (cognitive, emotional, and physiological) prior to the suicide action. A few patients describe a fast transition from suicidal impulses to the suicide action.

We hypothesized that focusing on the patients’ very personal narratives might be more helpful to meet the patients’ own understanding of a suicidal crisis than focusing on a psychiatric diagnosis [37,44]. In a clinical study based on single interviews shortly after a suicide attempt, we found that, in comparison to the usual clinical interviewing style, patients’ ratings of the therapeutic relationship in the Penn Helping Alliance Questionnaire [45] were significantly higher, when the interviewer in the introduction used a narrative approach. The approach was considered to be narrative when the interviewer used the words “tell” or “story” (“can you please tell me how you came to the point of harming yourself?” or “I would like to hear the story behind the suicidal crisis“) [46]. We discussed our findings in a conference with international experts of clinical suicidology, which developed into the Aeschi Working Group, and the biennial Aeschi Conferences [47]. The Guidelines for Clinicians formulated by this group emphasize that “the ultimate goal should be to engage the patient in a therapeutic relationship, even in a first assessment interview” [48]. From psychotherapy research, we know that the early therapeutic alliance is a major factor for therapy outcome [49,50,51].

A truly narrative approach requires newly defined roles of the patient and therapist: In the narrative, the patient is the “expert” of his or her suicide story, and the therapist is in a “not knowing position” [52] (p. 166); while in the psychiatric assessment of the patient’s mental state, the therapist is the expert. Following the narrative interview, we introduced a follow-up session, in which patients were confronted with the video-recorded interview. The video-playback and self-confrontation technique has been described by several authors [53,54,55]. In the video playback session, the patient and therapist sit side-by-side in front of the screen, watching the recorded interview; the prototype of a collaborative therapeutic approach [13]. The video is paused from time to time and patients are invited to report on any thoughts, feelings, and sensations that they had watching the interview, as well as to provide additional information regarding the suicide narrative. The aim of the video-playback is to achieve emotional and cognitive activation and restructuring, fostering self-awareness and insight, as the following letter exemplifies.

Dear doctor

Since I have seen you I have been feeling unburdened. Although about a week ago I experienced again something like beginning thoughts about suicide, I do feel better than three weeks ago, after the suicide attempt. Since then I also talked more with friends, and I tried again and again to explain what happened. I feel that the interview, and above all, watching together the video afterwards, gave me very much in terms of working through. Today it is much more clear to me what a “silly” idea such a suicide attempt, or suicide itself, is.

Again, many thanks! With best regards, R.W.

[56]

Although the patient’s view of a suicide attempt as something “silly” does not do justice to the traumatic dimension of an acute suicidal crisis, it indicates that, through the video playback, the patient has gained some insight into the altered mental state he experienced during the acute suicidal crisis. In action theoretical terms, the video playback technique is a joint action between patient and therapist, aimed at a shared goal: To understand the critical points of the suicidal process in the context of relevant biographical issues, to identify vulnerability and trigger factors, and to develop alternatives for future survival. Patient and therapist take an insider’s, as well as an outsider’s, position. In terms of mentalization [52], it is a process of joint attention, wherein the patient’s mental state is typically the focus of their shared attention, in a supportive and safe environment.

## 4. We Need Brief and Effective Therapies for Patients Who Attempt Suicide

“*Ensure that people who have attempted suicide can get effective interventions to prevent further attempts*”, Aspirational Goal Nr. 6, Research Agenda of the National Action Alliance of Suicide Prevention.

[57]

In contrast to the large number of suicidal individuals who do not seek professional help, those admitted to emergency departments following a suicide attempt enter the health care system, and can thus be taken into follow-up care. This is a key focus of clinical suicide prevention [58]. Attempted suicide is the main risk factor for suicide and suicide reattempts [59]. The suicide risk increases with each attempt and remains high over decades [60]. Repetition of non-fatal self-harm is common: Approximately 15%–25% of people who self-harm will repeat an episode within one year, and 20%–25% over the next few years [61]. Emergency department visits and inpatient hospitalizations due to suicidal ideation and suicide attempts, result in over 1 million hospital visits per year, leading to costs of some US$4.7 billion [62]. It has been estimated that interventions that reduce suicide attempts by 25% may lead to a 2.6% reduction in the suicide rate, which would result in approximately 1000 lives being saved in the US, annually [63].

Considering the limited resources for follow-up treatment of the large group of patients who attempt suicide, there is a clear need for brief and focused treatments for these patients [58,64]. Based on a model of suicide as a goal-directed action, we developed a brief therapy program for patients with a recent history of attempted suicide.

## 5. ASSIP (Attempted Suicide Short Intervention Program)

“*Being empathic with the suicidal wish means assuming the suicidal person’s perspective and ‘seeing’ how this person has reached a dead end without trying to interfere, stop, or correct the suicidal wishes. This means that the therapist attempts to empathize with the patient’s pain experience to such a point that he/she can ‘see’ why suicide is the only alternative available to the patient…Instead of working against the suicidal stream*”.

[65]

ASSIP is a treatment administered in three 60–90 min sessions, ideally within three weeks. A fourth session can be added if considered necessary.

### 5.1. First Session

A narrative interview is conducted, in which patients are asked to tell their personal stories about how they had reached the point of wanting to kill themselves, and how they went about it. The aim of the narrative interview is to reach—in a biographical context—a patient-centred understanding of the individual mechanisms leading to psychological pain and suicidal behaviour, and to elicit specific vulnerability factors and trigger events. All interviews are video-recorded, with the patients’ written consent.

### 5.2. Second Session

The patient and therapist watch selected sequences of the video-recorded interview, sitting side-by-side. Thus, the patient is put into the observer’s seat, watching the suicide narrative recorded in session one. The therapist helps to provide a detailed reconstruction of the transition from an experience of psychological pain and stress, to the suicidal action. Automatic thoughts, emotions, physiological changes, and contingent behaviour are identified. At the end of the session, two patients are given a psychoeducative handout (“Suicide is not a rational act”) as a homework task, to be returned, with personal comments, at the next session. The handout aims to establish a shared model of suicidal behaviour, by integrating theoretical concepts such as suicide risk factors, psychological pain, and the suicidal mode, as well as basic neurobiological correlates of the suicidal mind. Following the second session, the therapist prepares a written draft of the case conceptualization.

### 5.3. Third Session

The patients’ written feedback, in response to the handout, is discussed. The draft of the case conceptualization is collaboratively revised. The case conceptualization formulates personal vulnerabilities and suicide triggers, providing the rationale for the need to develop individual warning signs and safety strategies for future suicidal crises. The written case conceptualization and the personal safety strategies are printed and handed out to the patient, with additional copies for the health professionals involved in treatment. Long-term goals, warning signs, and safety strategies are copied to a credit-card sized folded leaflet and given to the patient. Patients are instructed to carry this leaflet on them at all times, and to consult it in the event of an emotional crisis.

### 5.4. Letters

Participants are sent semi-standardized letters over a period of 24 months, 3-monthly in the first year, and 6-monthly in the second year. The letters remind participants of the long-term risk of future suicidal crises and the importance of the safety strategies. Letters are signed personally by the ASSIP therapists. Patients are informed that they do not have to respond to the letters, but that a feedback about how things are going would be welcome. In the cases where patients write back (usually vie e-mail), the ASSIP therapist acknowledges this in the next letter.

For further details, see the ASSIP manual [56].

## 6. Evaluating ASSIP

“*ASSIP, a manual-based brief therapy for patients who have attempted suicide, administered in a real-world clinical setting, was efficacious in reducing suicidal behavior over 24 months. ASSIP thus fulfils the need for a brief, easy-to-implement, and low-cost intervention*”.

[66]

### 6.1. Method

Patients admitted to the emergency unit of the Bern University General Hospital following attempted suicide, were randomly allocated to treatment as usual (*N* = 60) or treatment as usual plus ASSIP (*N* = 60). ASSIP participants received three therapy sessions followed by regular contact through personalized letters over 24 months. Participants considered to be at high risk of suicide were included; 63% were diagnosed with an affective disorder and 50% had a history of prior suicide attempts. Clinical exclusion criteria were habitual self-harm, serious cognitive impairment, and psychotic disorder. The primary outcome measure was repeat suicide attempts during the 24-months follow-up period. Secondary outcome measures were suicidal ideation and healthcare utilization. Furthermore, the effects of prior suicide attempts, depression at the baseline, diagnosis, and therapeutic alliance on the outcome, were investigated.

### 6.2. Results

Treatment and control groups did not differ in demographic or clinical variables, with the exception of the number of outpatient sessions prior to the index suicide attempt. During the 24-months follow-up period, five repeat attempts were recorded in the ASSIP group and 41 attempts in the control group. The rates of participants reattempting suicide at least once were 8.3% (*n* = 5) and 26.7% (*n* = 16), respectively. ASSIP was associated with an approximately 80% reduced risk of repeat episodes (Wald χ^2^_1_ = 13.1; 95% CI: 12.4–13.7; *p* < 0.001). ASSIP participants spent significantly fewer days in hospital during the follow-up period. Higher therapeutic alliance in this group was associated with a lower rate of repeat attempts. Prior suicide attempts, depression, and a diagnosis of personality disorder at the baseline, did not significantly affect the outcome.

### 6.3. Conclusions

ASSIP, a manual-based brief therapy for patients who had recently attempted suicide, administered in addition to the usual clinical treatment, was effective in reducing suicidal behaviour in a real-world clinical setting. ASSIP fulfils the need for an easy to administer, low-cost intervention. Future studies with larger patient samples are required to determine the effectiveness of ASSIP in other clinical and sociocultural settings.

The study can be openly accessed at PLOS Medicine [66].

## 7. What Makes ASSIP Effective?

“*Nothing diminishes anxiety as much as a sense of how to proceed in an anxiety-provoking situation. Like our patients, we therapists can benefit from structure*”.

[67]

The finding that a very brief therapy can have a remarkable effect on the rate of repeat attempts over a time period of two years made us ask questions about the components that are likely to be effective in ASSIP. As ASSIP has been evaluated in the RCT as a “package”, we can only speculate about the therapy process factors involved.

### 7.1. Clear Structure and Treatment Goals

ASSIP is a highly structured treatment program, with clear objectives for each session, and is easy to understand for patients. Patients are informed about the treatment goals at the beginning of therapy. It is made clear that ASSIP will not “cure” suicidality, but that working together will lead to the tools required to respond differently to any future suicidal crisis. One of the main findings in psychotherapy research is that the outcome is associated with the patient-therapist agreement on treatment goals and the collaboration on treatment tasks necessary for goal attainment [68]. Furthermore, recent literature has highlighted the benefit of simple, well-structured, and, for patients, easy to understand therapy programs [69]. Brief treatments are typically patient-centred, facilitating a therapeutic alliance based on collaboration, information, and trust. This therapeutic approach stands in contrast to a relationship between a “healing” therapist and a passive patient [69]. In the treatment of people who attempt suicide, an increasing awareness of the mechanisms related to acute suicidality leads to a sense of empowerment, an improved sense of control, and a reduction of guilt [67], (p. 89); [70].

### 7.2. Therapeutic Alliance

Building a therapeutic alliance with the patient has been the key element in designing ASSIP. It is based on the belief that a patient-centred and collaborative approach is a must, in order to achieve a lasting therapeutic change. ASSIP has emerged from the work of the Aeschi Working Group, which has been called the “Aeschi-philosophy”. The essence of this therapeutic approach has been summarized in a multi-author volume [71]. Therapeutic alliance is understood as a process “in which patients allow a therapist to enter their personal world, in order to initiate a process of intrapsychic change. Once a therapeutic alliance has been established, a bond between the patient and therapist will ensue, which will often be lasting, and will continue beyond the formal termination of treatment“ [72].

### 7.3. Narrative Interviewing

A key element in ASSIP is the narrative interview. Suicidality is understood as a highly individual phenomenon, with a strong biographical background. The gold standard is to see “the patient’s percepts through the eyes of the patient” [73], (p. 46). We had hypothesized that a narrative approach, based on the notion that suicide is an action, would enhance therapeutic alliance—which would then be associated with a better therapy adherence and outcome. In the RCT, this hypothesis was supported by the finding that the dropout rate in the treatment group was very low (4/60), compared to the control group (13/60). In the treatment group, the therapeutic alliance measured with the Penn Helping Alliance Questionnaire (HAQ), increased from session one to session three, which we take as a sign that patients increasingly felt that the treatment was helpful. Furthermore, we found an inverse relationship between the HAQ scores in the first session and suicide ideation during the course of follow-up sessions [74]. Thus, the narrative interview establishes a joint life-enhancing project, in which there is a growing engagement with treatment.

### 7.4. Video-Playback and Self-Confrontation

The video-playback session is a powerful experience for patients, reviewing their own narratives in a safe and supportive environment. Being in the position of the observer is a form of self-confrontation with one’s own suicidal wish. The external mode is “a starting point for the real work of accessing and articulating felt emotions and elaborating new meanings” [75]. A theoretical model conceptualizing the process of the video self-confrontation is offered by the two-mode model of cognitive-emotional processing [76]. Stanovich and West [77] referred to these two systems as System 1 and System 2. System 1 in this theoretical frame operates with little or no conscious input, is intuitive and automatic, and is the source of most of our actions. Although usually reliable in familiar situations, with generally swift and appropriate responses to challenges, this computational system is subject to systematic errors in specific circumstances. It typically operates within an emotional context and has a tendency to jump to conclusions on the basis of very limited evidence [78]. System 2 has the function of an override system for risky decisions provided by System 1—such as planning and initiating suicidal behaviour. In this theoretical frame, the video self-confrontation can be seen as an activation of System 2, with patients reflecting on their suicide story and the possibility of other solutions than suicide to a seemingly unbearable state of mind. Furthermore, in ASSIP, the video self-confrontation prepares the ground for the motivation to detect future situations that require increased cognitive control [79].

### 7.5. Psychoeducative Handout

The handout given to patients is a four-page summary of several aspects of suicidal behaviour, fostering the patients’ cooperation with the homework task, which requires the provision of written feedback to the therapist. The goal is to collaboratively develop a model of suicidal behaviour, which will be informed by the therapist’s professional knowledge, integrating the patient’s experiential knowledge. The model of suicide presented in this handout (“Suicide is not a rational decision”) includes clinically relevant aspects of suicidal behaviour, such as psychological pain, the suicidal mode, and risk factors such as depression, early trauma, etc., as well as findings from neurobiological research.

*Psychological pain* is explained as the result of an experience that fundamentally threatens the sense of self. It explained that psychological pain is triggered by a negative experience, such as a threatened or actual breakdown of a relationship, or by an experience of personal failure or loss of important personal goals. “This pain may be worse than the most extreme physical pain. When we see no solution to such a painful experience, a state of alarm will ensue, which may be difficult to control. We lose faith that this experience of alarm and intense pain will ever subside.” The handout explains that dissociative symptoms are typical for the experience of extreme psychological pain: “Suicidal people report that they were not their usual self anymore and that they were acting in a trance-like state, that they felt disconnected from their physical body, and felt no pain. These critical mental states are called “dissociation”, which means that the normal self-perception is disrupted. In such a condition it is practically impossible to think and act rationally.” Dissociative symptoms related to psychological pain have been described by Orbach [80,81]. This concept, in our experience, is extremely helpful for patients to reduce the shame that is often associated with self-harm.

*The suicidal mode* has been described as a cognitive-emotional-behavioral state of mind, designed to deal with specific, extraordinary situations, such as emotionally stressful or traumatic experiences. In ASSIP, it is described as an on/off phenomenon, which can be reactivated at any time [70,82]. The handout introduces findings from brain research: “After the recent suicidal crisis the suicidal mode will be stored in your brain circuitry and be readily available in future similar situations”. It is explained that, in the acute suicidal crisis, the neural activity in the brain region responsible for problem solving, i.e., the prefrontal cortex, is deactivated [83,84], and that therefore, safety strategies need to be developed. These must be implemented before the suicidal mode is fully activated.

### 7.6. Safety Planning

**Figure 1:**
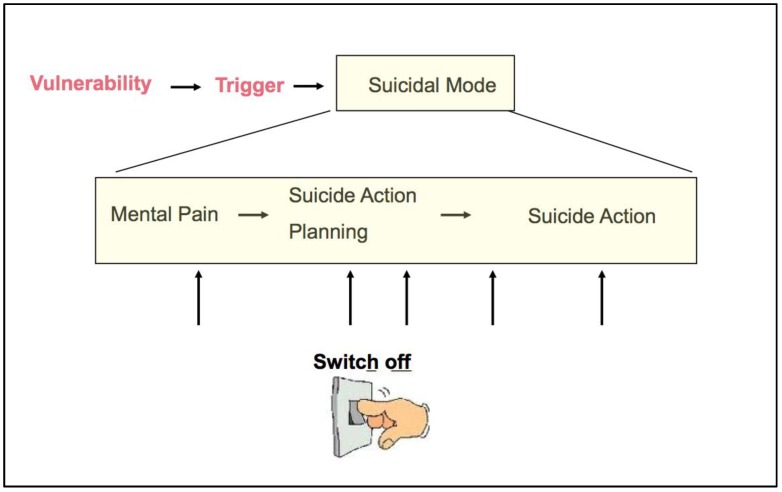
The treatment model of ASSIP, illustrating that safety strategies can switch off the suicidal mode at various stages in the development of the suicidal mode.

Safety planning is a therapeutic concept well known from CBT [16,85]. It is closely related to the concept of the suicidal mode, which, in ASSIP, is broken down into different stages of the suicidal development in which we differentiate between the mental pain phase, the suicide action planning, and the phase of suicide action execution. This model demonstrates to the patient that safety strategies can be put into action at various stages (see Figure 1). These include first stage strategies, in which patients can use their own personal resources, and second stage acute strategies, that involve personal and professional helpers or institutions. Early warning signs and safety strategies are collaboratively determined.

### 7.7. Written Case Conceptualization

The draft of the case conceptualization, prepared by the therapist, is revised sentence-by-sentence in close collaboration with the patient, so that the patient “owns” this summary of the suicidal crisis. Most importantly, the text includes the patient’s specific vulnerabilities and the events triggering the suicidal action—factors that, in the future, could again activate the suicidal mode. The case conceptualization prepares the ground for formulating warning signs and safety strategies.

### 7.8. Outreach Elements

Apart from home visits and phone calls, the most common outreach elements are regular letters and postcards [86,87,88,89,90]. Although their preventive effect has been mixed, we felt that personalised letters following the face-to-face sessions would mean more to the patients than anonymous letters: “A “lifeline” is only helpful if there is someone at the other end, i.e., first there has to be a trustful relationship” [91]. In ASSIP, the therapist continues to be a representation of a “secure base” experience in the sense of attachment theory [92]. The notion of the secure base states that, “in times of calamity”, humans seek the support of a person that understands them and can provide security. The letters function as a prolongation of a meaningful therapeutic relationship, as well as reminders of the collaboratively developed safety strategies. The crisis card, a credit-card sized, folded list of personalized safety strategies, serves the same purpose.

## 8. Lessons Learnt from ASSIP: Can They Be Used to Improve Concepts of Suicide Prevention?

“*… suicidology has disproportionately focused on explaining suicide rather than understanding it. We need to better understand the particular circumstances associated with better outcomes*”.

[93]

Suicide prevention is faced with many barriers, and we need to improve the ways in which we can overcome them. Thoughts of suicide once or several times in a lifetime are common in Western societies—[94]. However, as described in Section 2 of this article, suicidal thoughts and plans are often not addressed in medical consultation. Suicidal persons suffer from a loss of self-esteem, and they are ashamed of not being able to cope with their problems. They are afraid of not being understood. They are afraid of stigmatisation. To be told that they suffer from a depression may be helpful for some of them, but for others, it makes things worse. Men in particular tend to see the diagnosis of depression as yet another failure in their life [95]. Patients and therapists need concepts that help them to overcome their helplessness vis-à-vis the phenomenon of suicidal behaviour. Concepts used in suicide prevention must provide a common conceptual ground, where suicidal individuals and health professionals can meet. ASSIP uses some novel concepts of suicidal behaviour and treatment, which may have the potential to be effective elements in other areas of suicide prevention. Here, we try to formulate the lessons learnt from ASSIP in a general manner.

### 8.1. Concept Nr. 1: Suicide Is an Action and Has a Personal History

Affective disorders and other psychiatric diagnoses are risk factors for suicide, not the cause. Understanding suicide as an action shifts the focus to the individuality of suicidal behaviour. Every suicide story is different. Suicidal behaviour is not the result of a single cause. Unfortunately, in clinical practice, particularly in medical and psychiatric institutions, the clinicians’ listening abilities have often been replaced by questionnaires and manualized interviews. Yet, a “one-size-fits all approach” will not reach the suicidal individual [96]. People with suicidal ideation will only open up and confide their stories of pain and shame if the therapist is an empathic listener, who wants to learn from and understand “the person in the patient” [97]. Suicidal individuals have an impressive narrative competence; that is, they are well able to provide a congruent story. Once a person feels understood and has developed trust, the path will be open for a truly collaborative therapy. The guidelines for clinicians issued by the Aeschi Working Group, state that “an empathic approach is essential to help patients re-establish life-oriented goals” [48]. To listen to patients in an empathic, attentive, and nonjudgmental way, is, of course, not something new; it is simply good clinical practice.

### 8.2. Concept Nr. 2: Suicide Happens in an Altered State of Consciousness

Suicide and attempted suicide are usually the result of adverse events leading to an emotional crisis, best characterized by the concept of psychological pain. Psychological pain and the feeling of hopelessness lead to the decision that suicide is the only way left to end the suffering. This may be so convincing that alternatives, or life-oriented plans and goals, are out of focus. The suicidal brain is focused on a quick solution for the unbearable state of mind, which seems fully convincing in that given moment (“cognitive-emotional myopia”). A person who cannot simulate alternatives to a given situation becomes “stimulus bound”. Patients with a history of suicidal behaviour have impaired decision-making capacities [98], a finding associated with reduced activity in frontal brain areas [99]. Dissociative symptoms, which in clinical practice are often not recognized, point to a mental condition with trauma-like dimensions [100]. An fMRI study using script-driven recall of the suicidal crisis, suggested that changes in the prefrontal neural activity have a strong state-dependent component [83]. In the suicidal mode characterized by dissociative symptoms, the plan is put into action in the sense of an “auto-pilot” condition.

### 8.3. Concept Nr. 3: In a Crisis We Cannot Always Trust Our Brains

We contend that suicide is an action that, in retrospect, most people would regret. Patients who survive a suicidal act, typically realize that alternatives would have been available, and that in retrospect, suicide, in view of their long-term life plans, was a wrong decision. This is convincingly demonstrated by Kevin Hines, who talks about his jump from the Golden Gate Bridge [101]. The model of suicide as an action related to seriously impaired decision-making, reduces stigma and shame; the latter being one of the main barriers to people who need to seek help [12,41]. Therefore, the message in a prevention campaign could be: “In an emotional crisis be aware: you cannot always trust your brain. Suicide is an action that your brain may convincingly see as the only solution. It is not a psychiatric disorder, and there is no reason to be ashamed. It can happen to anybody. It is time-limited. There is always another solution. You should discuss your condition with a professional helper. Your life is too precious.”

### 8.4. Concept Nr. 4: After a Suicidal Crisis: Beware of the Increased Suicide Risk

We believe that, after a suicide attempt, suicidality cannot be “cured” for good. Research suggests that the suicidal mode is also a neurobiological mode. It is stored in the neural circuitry and will be triggered again in the future by adverse personal experiences. After a suicidal crisis, people need to be aware of the increased risk. They need to develop an awareness of the warning signs, and use safety strategies during an early stage in the development of the suicidal mode. Psychoeducation is key for clinical suicide prevention. Furthermore, outreach elements such as regular letters, phone calls, and home visits appear to be useful preventive measures. The effect of psychoeducation and continued contact has been demonstrated in the Fleischman et al. [102] study. A personal, long-term follow-up by the same health professional (as in ASSIP) appears to be particularly helpful [103]. This may also account for the beneficial effect of long-term pharmacotherapy, which requires a long-term relationship with a health professional [104].

## 9. Conclusions

“*No single initiative will suffice; rather, there must be comprehensive, integrated efforts that reach across health systems and communities, (...). Mosaics, where pieces fit together to make a more complete picture, are required to address the extraordinary diversity of those who die by suicide*”.

[105]

ASSIP, a brief and effective therapy for patients seen after a suicide attempt, introduced some novel therapeutic elements such as narrative interviewing, video-playback, and case conceptualizations, using the concept of suicide as a goal-directed action. In this article, it has been argued that theoretical concepts on which ASSIP is based, may be useful for new clinical and universal prevention projects. These concepts also imply certain future directions in suicide research:

- Neurobiological research has not “found” the cause of suicide, but it has given us exciting insights into the brain and the brain function related to suicidal behaviour [106,107,108]. We need more research focusing on the neurobiology of actions and decision-making, both trait- and state-dependent.
- We need to understand the relationship between problems of decision-making, psychiatric pathology, and childhood trauma.
- We need large randomized treatment trials in various clinical settings, with long-term follow-up.
- We need more psychotherapy research to find out which therapy process factors are most effective in the treatment of suicidality. There is a huge need for brief and effective therapies.
- We need to investigate the effect of successful therapies on brain function.
- On universal and indicated levels of prevention, we need to learn which models of suicide best reach people, in order to change attitudes and knowledge about suicide, to reduce stigma and shame, and to increase help-seeking behaviour.

## References

[1] Maris R. (1981). Pathways to Suicide: A Survey of Self-Destructive Behaviors.

[2] Durkheim E. (1897). Le suicide: Etude de Sociologie (Suicide: A Sociological Study).

[3] Mann J.J., McBride P.A., Brown R.P., Linnoila M., Leon A.C., DeMeo M., Mieczkowski T., Myers J.E., Stanley M. (1992). Relationship between central and peripheral serotonin indexes in depressed and suicidal psychiatric inpatients. Arch. Gen. Psychiatry.

[4] Rihmer Z. (2007). Suicide risk in mood disorders. Curr. Opin. Psychiatry.

[5] Borges G., Nock M.K., Haro Abad J.M., Hwang I., Sampson N.A., Alonso J., Andrade L.H., Angermeyer M.C., Beautrais A., Bromet E. (2010). Twelve-month prevalence of and risk factors for suicide attempts in the world health organization world mental health surveys. J. Clin. Psychiatry.

[6] De Leo D. (2002). Why are we not getting any closer to preventing suicide?. Br. J. Psychiatry.

[7] Bertolote J.M., Fleischmann A., De Leo D., Wasserman D. (2003). Suicide and mental disorders: Do we know enough?. Br. J. Psychiatry.

[8] Kapusta N.D., Niederkrotenthaler T., Etzersdorfer E., Voracek M., Dervic K., Jandl-Jager E., Sonneck G. (2009). Influence of psychotherapist density and antidepressant sales on suicide rates. Acta Psychiatr. Scand..

[9] Pacchiarotti I., Bond D.J., Baldessarini R.J., Nolen W.A., Grunze H., Licht R.W., Post R.M., Berk M., Goodwin G.M., Sachs G.S. (2013). The international society for bipolar disorders (ISBD) task force report on antidepressant use in bipolar disorders. Am. J. Psychiatry.

[10] Van Praag H.M. (2003). A stubborn behaviour: The failure of antidepressants to reduce suicide rates. World J. Biol. Psychiatry.

[11] Cipriani A., Hawton K., Stockton S., Geddes J.R. (2013). Lithium in the prevention of suicide in mood disorders: Updated systematic review and meta-analysis. BMJ.

[12] De Leo D. (2004). Suicide prevention is far more than a psychiatric business. World Psychiatry.

[13] Jobes D.A. (2000). Collaborating to prevent suicide: A clinical-research perspective. Suicide Life Threat. Behav..

[14] Linehan M.M. (2008). Suicide intervention research: A field in desperate need of development. Suicide Life Threat. Behav..

[15] Hjelmeland H., Knizek B.L. (2010). Why we need qualitative research in suicidology. Suicide Life Threat. Behav..

[16] Brown G.K., Ten Have T., Henriques G.R., Xie S.X., Hollander J.E., Beck A.T. (2005). Cognitive therapy for the prevention of suicide attempts: A randomized controlled trial. JAMA.

[17] Rudd M.D., Bryan C.J., Wertenberger E.G., Peterson A.L., Young-McCaughan S., Mintz J., Williams S.R., Arne K.A., Breitbach J., Delano K. (2015). Brief cognitive-behavioral therapy effects on post-treatment suicide attempts in a military sample: Results of a randomized clinical trial with 2-year follow-up. Am. J. Psychiatry.

[18] Linehan M.M., Armstrong H.E., Suarez A., Allmon D., Heard H.L. (1991). Cognitive-behavioral treatment of chronically parasuicidal borderline patients. Arch. Gen. Psychiatry.

[19] Jobes D.A. (2012). The collaborative assessment and management of suicidality (CAMS): An evolving evidence-based clinical approach to suicidal risk. Suicide Life Threat. Behav..

[20] Zalsman G., Hawton K., Wasserman D., van Heeringen K., Arensman E., Sarchiapone M., Carli V., Hoschl C., Barzilay R., Balazs J. (2016). Suicide prevention strategies revisited: 10-Year systematic review. Lancet Psychiatry.

[21] Hetrick S.E., Robinson J., Spittal M.J., Carter G. (2016). Effective psychological and psychosocial approaches to reduce repetition of self-harm: A systematic review, meta-analysis and meta-regression. BMJ Open.

[22] Michel K., Dey P., Valach L., Heeringen K.V. (2001). Suicide as goal-directed action. Understanding Suicidal Behaviour: The Suicidal Process Approach to Research and Treatment.

[23] Downs M.F., Eisenberg D. (2012). Help seeking and treatment use among suicidal college students. J. Am. Coll. Health.

[24] Michel K., Valach L., Waeber V. (1994). Understanding deliberate self-harm: The patients’ views. Crisis.

[25] Renaud J., Berlim M.T., Seguin M., McGirr A., Tousignant M., Turecki G. (2009). Recent and lifetime utilization of health care services by children and adolescent suicide victims: A case-control study. J. Affect. Disord..

[26] Isometsä E.T., Heikkinen M.E., Marttunen M.J., Henriksson M.M., Aro H.M., Lönnqvist J.K. (1995). The last appointment before suicide: Is suicide intent communicated?. Am. J. Psychiatry.

[27] Pirkis J., Burgess P. (1998). Suicide and recency of health care contacts. A systematic review. Br. J. Psychiatry.

[28] Wolk-Wasserman D. (1987). Contacts of suicidal neurotic and prepsychotic/psychotic patients and their significant others with public care institutions before the suicide attempt. Acta Psychiatr. Scand..

[29] Apter A., Horesh N., Gothelf D., Graffi H., Lepkifker E. (2001). Relationship between self-disclosure and serious suicidal behavior. Compr. Psychiatry.

[30] Murphy G.E. (1975). The physician’s responsibility for suicide. II. Errors of omission. Ann. Intern. Med..

[31] Shea S. (2004). The delicate art of eliciting suicidal ideation. Psychiatr. Ann..

[32] Michel K. (1986). Suicide and suicide prevention. Could the physician do more? Results of a questionnaire of relatives of suicide attempters and suicide victims. Schweiz. Med. Wochenschr..

[33] Kurz A., Möller H.J. (1984). Help-seeking behavior and compliance of suicidal patients. Psychiatr. Prax..

[34] Fawcett J., Scheftner W.A., Fogg L. (2001). Predictive factors of post-discharge follow-up care among adolescent suicide attempters. Acta Psychiatr. Scand..

[35] Monti K., Cedereke M., Ojehagen A. (2003). Treatment attendance and suicidal behavior 1 month and 3 months after a suicide attempt: A comparison between two samples. Arch. Suicide Res..

[36] Treolar A.J., Pinfold T.J. (1993). Deliberate self-harm: An assessment of patients’ attitudes to the care they receive. Crisis.

[37] Michel K., Valach L. (1997). Suicide as goal-directed action. Arch. Suicide Res..

[38] Gollwitzer P.M., Gollwitzer P.M., Bargh J.A. (1996). The volitional benefits of planning. The Psychology of Action. Linking Cognition and Motivation to Behavior.

[39] Valach L., Michel K., Young R.A., Dey P., Valach L., Young R.A., Lynam M.J. (2002). Attempted suicide stories: Suicide career, suicide project and suicide action. Action Theory Primer for Applied Research in the Social Sciences.

[40] Maltsberger J.T. (2004). The descent into suicide. Int. J. Psychoanal..

[41] Tornblom A.W., Werbart A., Rydelius P.A. (2013). Shame behind the masks: The parents’ perspective on their sons’ suicide. Arch. Suicide Res..

[42] Valach L., Michel K., Young R.A. (2016). Suicide as a distorted goal-directed process: Wanting to die, killing, and being killed. J. Nerv. Ment. Dis..

[43] Adler H.M. (1997). The history of the present illness as treatment: Who’s listening, and why does it matter?. J. Am. Board Fam. Pract..

[44] Valach L., Young R.A., Lynam M.J. (2002). Action Theory: A Primer for Applied Research in the Social Sciences.

[45] Alexander L.B., Luborsky L., Greenberg L.S., Pinsoff W.M. (1986). The penn helping alliance scales. The Psychotherapeutic Process: A Research Handbook.

[46] Michel K., Dey P., Stadler K., Valach L. (2004). Therapist sensitivity towards emotional life-career issues and the working alliance with suicide attempters. Arch. Suicide Res..

[47] The Aeschi Working Group Meeting the Suicidal Person. http://www.aeschiconference.unibe.ch/.

[48] Michel K., Maltsberger J.T., Jobes D.A., Leenaars A.A., Orbach I., Stadler K., Dey P., Young R.A., Valach L. (2002). Discovering the truth in attempted suicide. Am. J. Psychother..

[49] Horvath A.O., Symonds B.D. (1991). Relation between working alliance and outcome in psychotherapy: A meta-analysis. J. Couns. Psychol..

[50] Saltzman C., Luetgert M.J., Roth C.H., Creaser J., Howard L. (1976). Formation of a therapeutic relationship: Experiences during the initial phase of psychotherapy as predictors of treatment duration and outcome. J. Consult. Clin. Psychol..

[51] Zuroff D.C., Blatt S.J., Sotsky S.M., Krupnick J.L., Martin D.J., Sanislow C.A., Simmens S. (2000). Relation of therapeutic alliance and perfectionism to outcome in brief outpatient treatment of depression. J. Consult. Clin. Psychol..

[52] Allen J.G., Fonagy P., Bateman A. (2008). Mentalizing in Clinical Practice.

[53] Hermans H.J., Hermans-Jansen E. (1995). Self-Narratives.

[54] Young R.A., Valach L., Dillabough J.-A., Dover C., Matthes G. (1994). Career research from an action perspective: The self-confrontation procedure. Career Dev. Q..

[55] Valach L., Michel K., Dey P., Young R.A. (2002). Self-confrontation interview with suicide attempters. Couns. Psychol. Q..

[56] Michel K., Gysin-Maillart A. (2015). ASSIP—Attempted Suicide Short Intervention Program. A Manual for Clinicians.

[57] National Institute of Mental Health and the Research Prioritization Task Force (2014). A Prioritized Research Agenda for Suicide Prevention: An Action Plan to Save Lives.

[58] Lizardi D., Stanley B. (2010). Treatment engagement: A neglected aspect in the psychiatric care of suicidal patients. Psychiatr. Serv..

[59] Runeson B.S. (2002). Suicide after parasuicide. BMJ.

[60] Hawton K., Zahl D., Weatherall R. (2003). Suicide following deliberate self-harm: Long-term follow-up of patients who presented to a general hospital. Br. J. Psychiatry.

[61] Owens D., Horrocks J., House A. (2002). Fatal and non-fatal repetition of self-harm systematic review. Br. J. Psychiatry.

[62] Yang B., Lester D. (2006). Recalculating the economic cost of suicide. Death Stud..

[63] Lewis G., Hawton K., Jones P. (1997). Strategies for preventing suicide. Br. J. Psychiatry.

[64] Arensman E., Townsend E., Hawton K., Bremner S., Feldman E., Goldney R., Gunnell D., Hazell P., Van Heeringen K., House A. (2001). Psychosocial and pharmacological treatment of patients following deliberate self-harm: The methodological issues involved in evaluating effectiveness. Suicide Life Threat. Behav..

[65] Orbach I. (2001). Therapeutic empathy with the suicidal wish: Principles of therapy with suicidal individuals. Am. J. Psychother..

[66] Gysin-Maillart A., Schwab S., Soravia L., Megert M., Michel K. (2016). A novel brief therapy for patients who attempt suicide: A 24-months follow-up randomized controlled study of the attempted suicide short intervention program (ASSIP). PLoS Med..

[67] Allen J., Michel K., Jobes D.A. (2011). Mentalizing suicidal states. Building a Therapeutic Alliance with the Suicidal Patient.

[68] Arnow B.A., Steidtmann D. (2014). Harnessing the potential of the therapeutic alliance. World Psychiatry.

[69] Colom F. (2011). Keeping therapies simple: Psychoeducation in the prevention of relapse in affective disorders. Br. J. Psychiatry.

[70] Rudd M.D. (2004). Cognitive therapy for suicidality: An integrative, comprehensive, and practical approach to conceptualization. J. Contemp. Psychother..

[71] Michel K., Jobes D.A. (2011). Building a Therapeutic Alliance with the Suicidal Patient.

[72] Michel K., O’Connor R.C., Pirkis J. (2016). Therapeutic alliance and the therapist. The International Handbook of Suicide Prevention.

[73] Jobes D.A. (2006). Managing Suicidal Risk: A Collaborative Approach.

[74] Gysin-Maillart A.C., Soravia L.M., Gemperli A., Michel K. (2016). Suicide ideation is related to therapeutic alliance in a brief therapy for attempted suicide. Arch. Suicide Res..

[75] Angus L., Levitt H., Hardtke K. (1999). The narrative processes coding system: Research applications and implications for psychotherapy practice. J. Clin. Psychol..

[76] Epstein S., Lipson A., Holstein C., Huh E. (1992). Irrational reactions to negative outcomes: Evidence for two conceptual systems. J. Pers. Soc. Psychol..

[77] Stanovich K.E., West R.F. (2000). Individual differences in reasoning: Implications for the rationality debate?. Behav. Brain Sci..

[78] Kahneman D. (2011). Thinking Fast and Slow.

[79] Botvinick M.M., Cohen J.D., Carter C.S. (2004). Conflict monitoring and anterior cingulate cortex: An update. Trends Cogn. Sci..

[80] Orbach I. (1994). Dissociation, physical pain, and suicide: A hypothesis. Suicide Life Threat. Behav..

[81] Orbach I. (2003). Suicide and the suicidal body. Suicide Life Threat. Behav..

[82] Beck A.T., Salkovskis P. (1996). Beyond belief: A theory of modes, personality, and psychopathology. Frontiers of Cognitive Therapy.

[83] Reisch T., Seifritz E., Esposito F., Wiest R., Valach L., Michel K. (2010). An fmri study on mental pain and suicidal behavior. J. Affect. Disord..

[84] Michel K., Michel K., Jobes D.A. (2011). Neurobiology and patient-oriented models of suicide—A contradiction?. Building a Therapeutic Alliance with the Suicidal Patient.

[85] Stanley B., Brown G.K. (2012). Safety planning intervention: A brief intervention to mitigate suicide risk. Cogn. Behav. Pract..

[86] Carter G.L., Clover K., Whyte I.M., Dawson A.H., D’Este C. (2005). Postcards from the edge project: Randomised controlled trial of an intervention using postcards to reduce repetition of hospital treated deliberate self poisoning. BMJ.

[87] Carter G.L., Clover K., Whyte I.M., Dawson A.H., D’Este C. (2013). Postcards from the edge: 5-Year outcomes of a randomised controlled trial for hospital-treated self-poisoning. Br. J. Psychiatry.

[88] Motto J.A., Bostrom A.G. (2001). A randomized controlled trial of postcrisis suicide prevention. Psychiatr. Serv..

[89] Beautrais A.L., Gibb S.J., Faulkner A., Fergusson D.M., Mulder R.T. (2010). Postcard intervention for repeat self-harm: Randomised controlled trial. Br. J. Psychiatry.

[90] Bennewith O., Evans J., Donovan J., Paramasivan S., Owen-Smith A., Hollingworth W., Davies R., O’Connor S., Hawton K., Kapur N. (2014). A contact-based intervention for people recently discharged from inpatient psychiatric care: A pilot study. Arch. Suicide Res..

[91] Hepp U., Wittmann L., Schnyder U., Michel K. (2004). Psychological and psychosocial interventions after attempted suicide: An overview of treatment studies. Crisis.

[92] Bowlby J. (1988). A Secure Base. Clinical Applications of Attachment Theory.

[93] O’Connor R., Platt S., Gordon J., O’Connor R., Platt S., Gordon J. (2011). Achievements and Challenges in Suicidology: Conclusions and Future Directions. International Handbook of Suicide Prevention.

[94] Kessler R.C., Borges G., Walters E.E. (1999). Prevalence of and risk factors for lifetime suicide attempts in the national comorbidity survey. Arch. Gen. Psychiatry.

[95] Rutz W., von Knorring L., Pihlgren H., Rihmer Z., Walinder J. (1995). Prevention of male suicides: Lessons from gotland study. Lancet.

[96] Rogers J.R., Soyka K.M. (2004). “One size fits all”: An existential-constructivist perspective on the crisis intervention approach with suicidal individuals. J. Contemp. Psychother..

[97] Sabo A.N., Rand B. (2000). The relational aspects of psychopharmacology. The Real World Guide to Psychotherapy Practice.

[98] Jollant F., Bellivier F., Leboyer M., Astruc B., Torres S., Verdier R., Castelnau D., Malafosse A., Courtet P. (2005). Impaired decision making in suicide attempters. Am. J. Psychiatry.

[99] Jollant F., Lawrence N.S., Olie E., O’Daly O., Malafosse A., Courtet P., Phillips M.L. (2010). Decreased activation of lateral orbitofrontal cortex during risky choices under uncertainty is associated with disadvantageous decision-making and suicidal behavior. Neuroimage.

[100] Maltsberger J.T., Leenaars A.A. (1993). Confusions of the body, the self, and others in suicidal states. Suicidology: Essays in Honour of Edwin S. Shneidman.

[101] Hines K. The Kevin Hines Story. http://www.kevinhinesstory.com/.

[102] Fleischmann A., Bertolote J.M., Wasserman D., De Leo D., Bolhari J., Botega N.J., De Silva D., Phillips M., Vijayakumar L., Varnik A. (2008). Effectiveness of brief intervention and contact for suicide attempters: A randomized controlled trial in five countries. Bull. World Health Organ..

[103] Sinclair J., Green J. (2005). Understanding resolution of deliberate self harm: Qualitative interview study of patients’ experiences. BMJ.

[104] Angst J., Angst F., Gerber-Werder R., Gamma A. (2005). Suicide in 406 mood-disorder patients with and without long-term medication: A 40 to 44 years’ follow-up. Arch. Suicide Res..

[105] Lytle M.C., Silenzio V.M., Caine E.D. (2016). Are there still too few suicides to generate public outrage?. JAMA Psychiatry.

[106] Mann J.J. (2003). Neurobiology of suicidal behaviour. Nat. Rev. Neurosci..

[107] Jollant F., Lawrence N.L., Olie E., Guillaume S., Courtet P. (2011). The suicidal mind and brain: A review of neuropsychological and neuroimaging studies. World J. Biol. Psychiatry.

[108] Turecki G., Ernst C., Jollant F., Labonte B., Mechawar N. (2012). The neurodevelopmental origins of suicidal behavior. Trends Neurosci..

